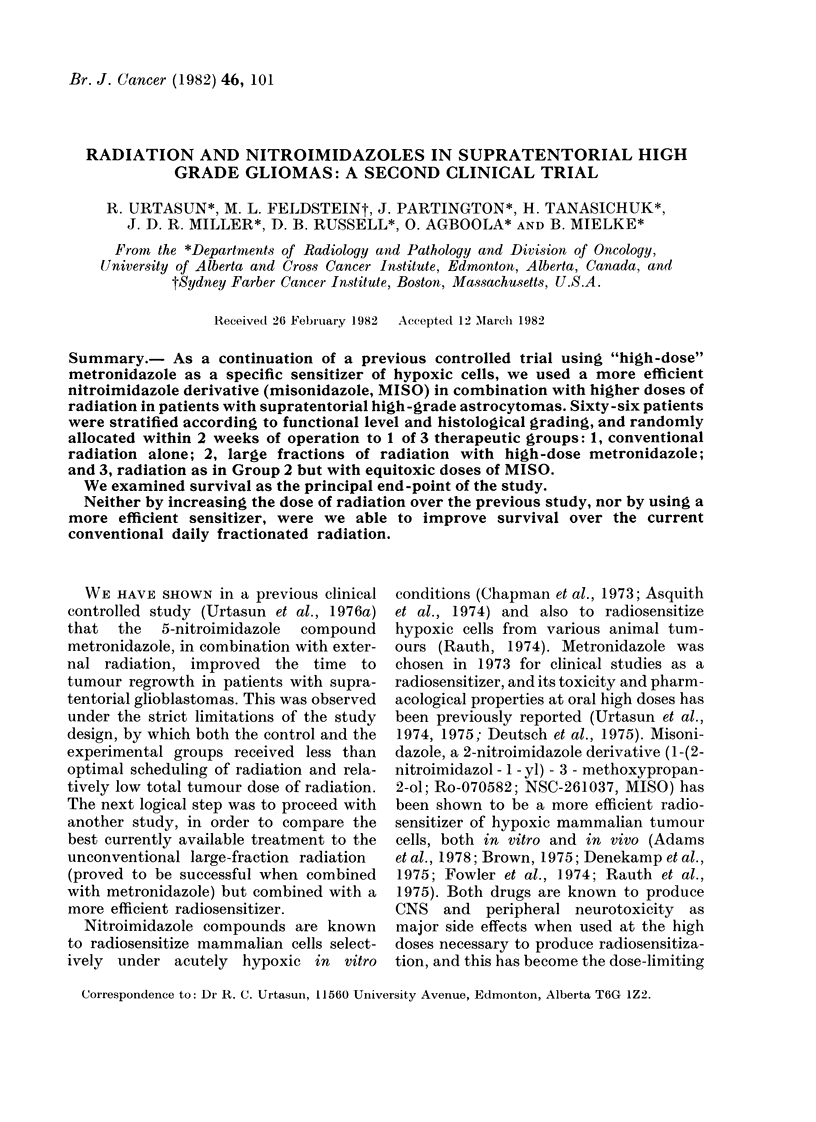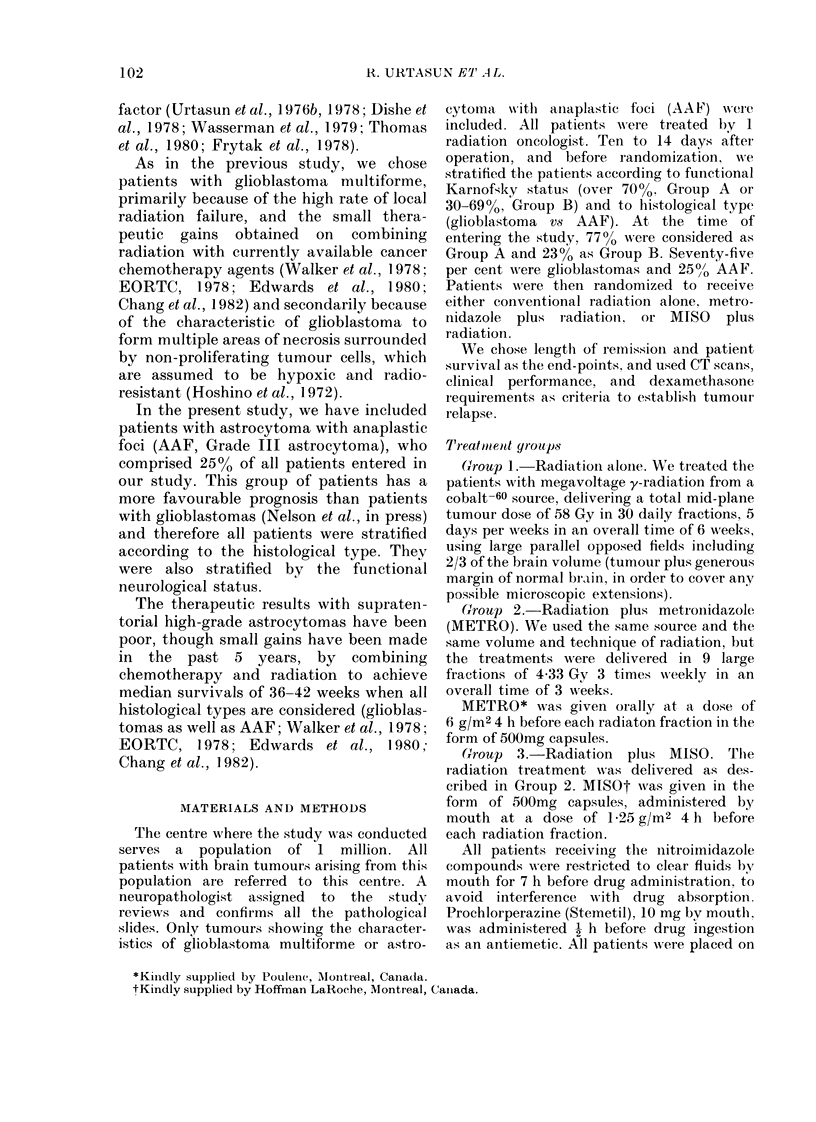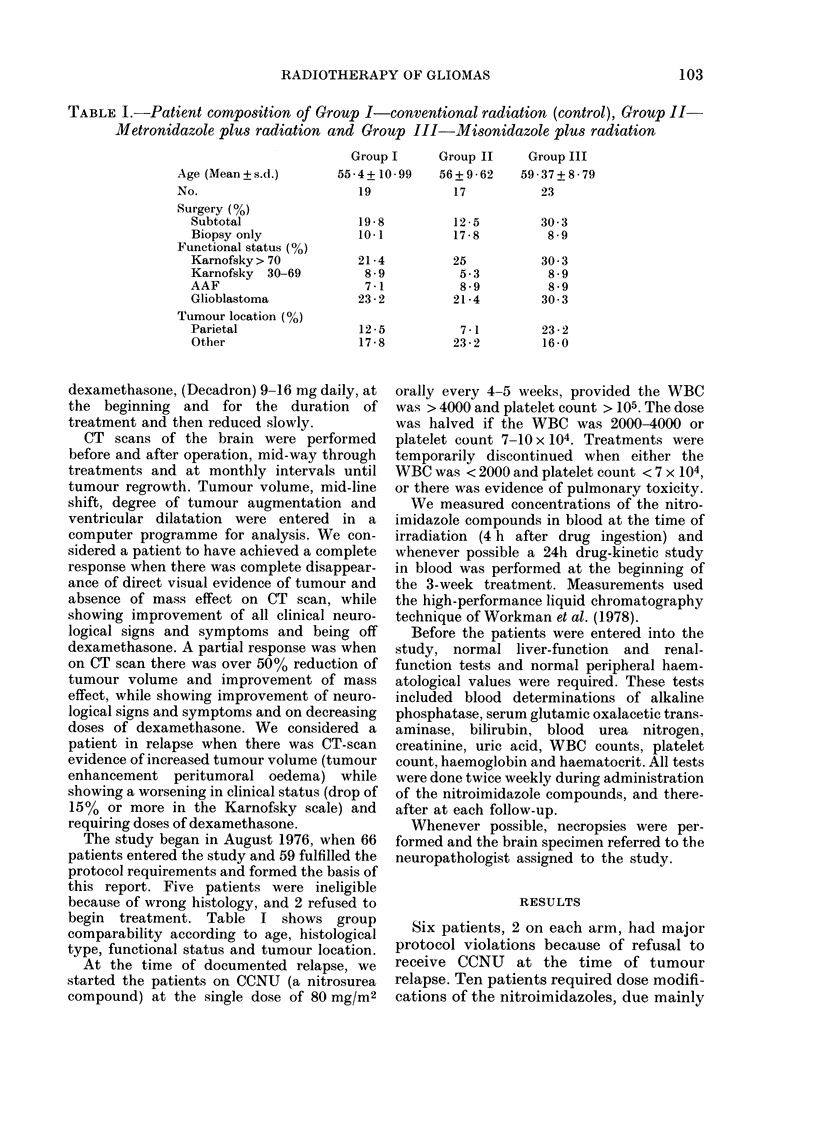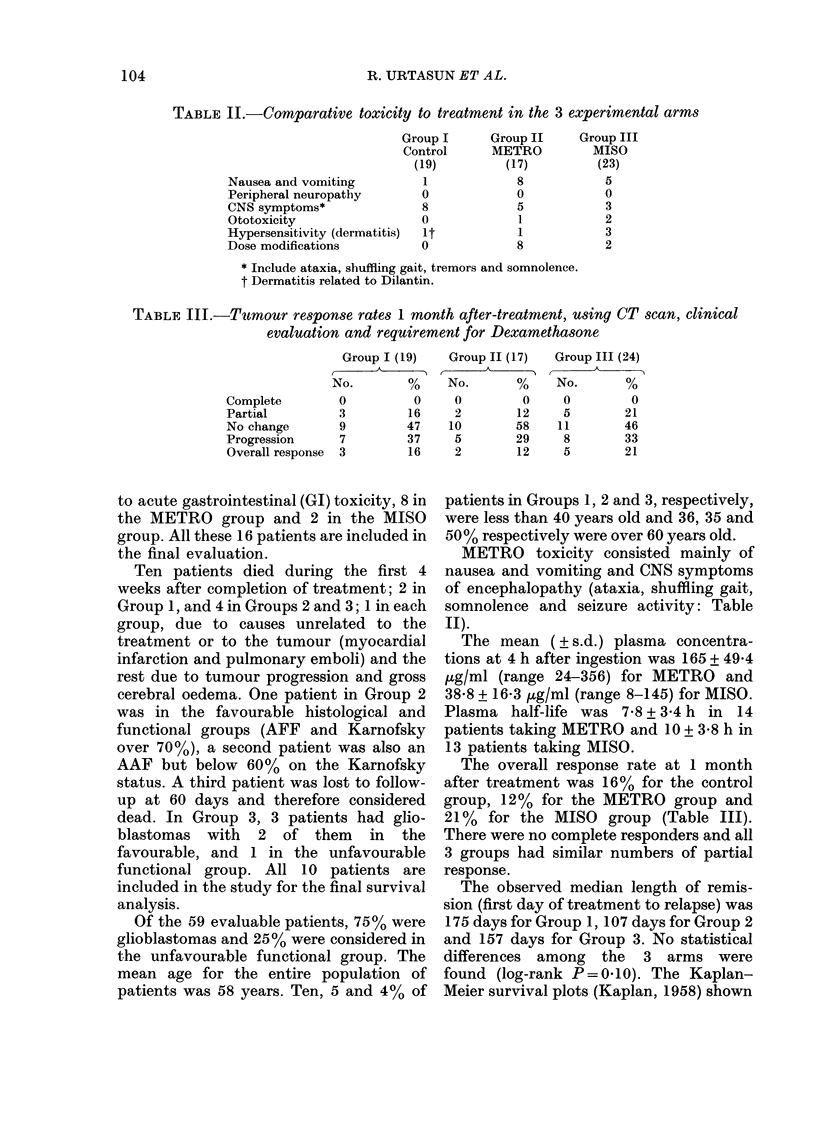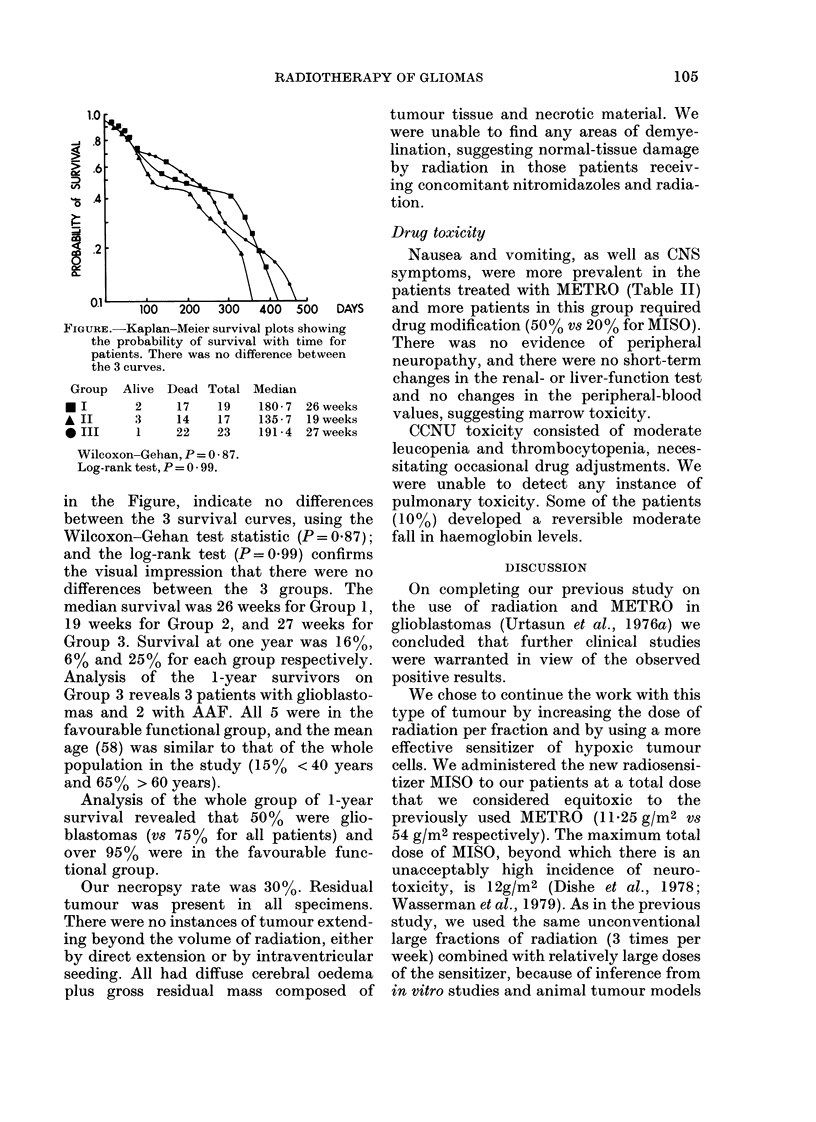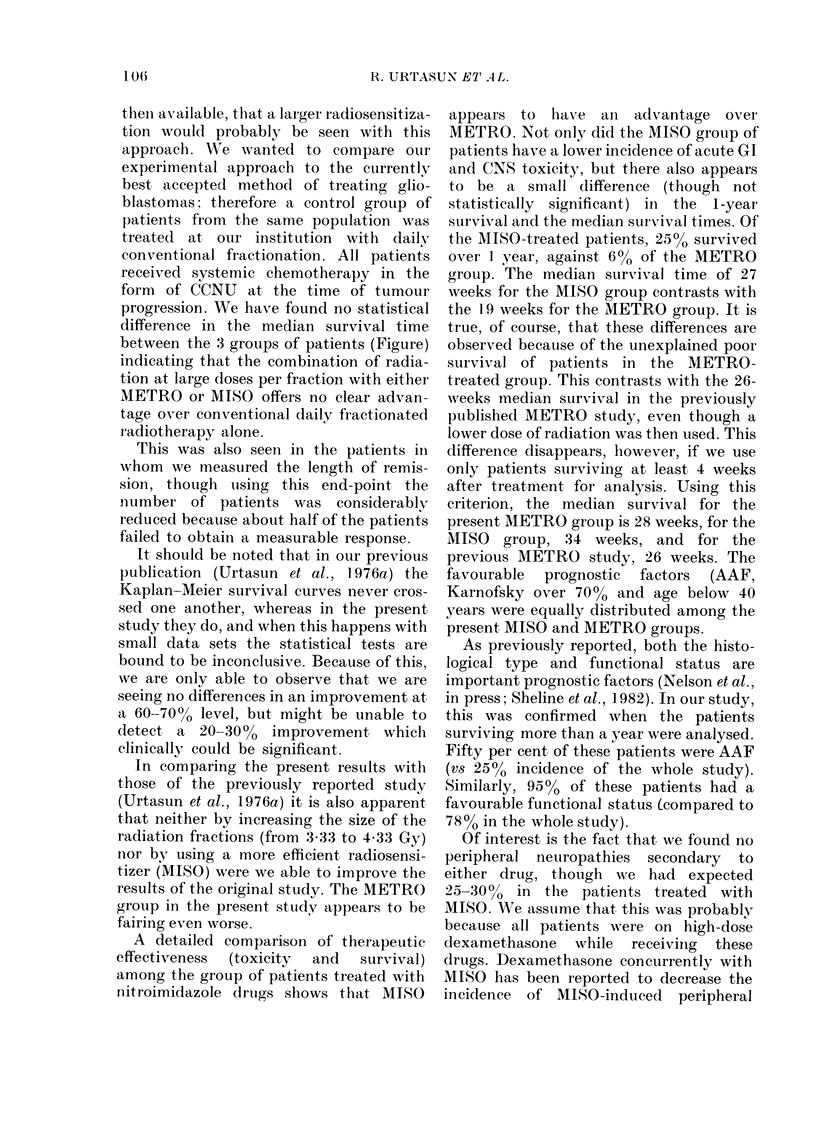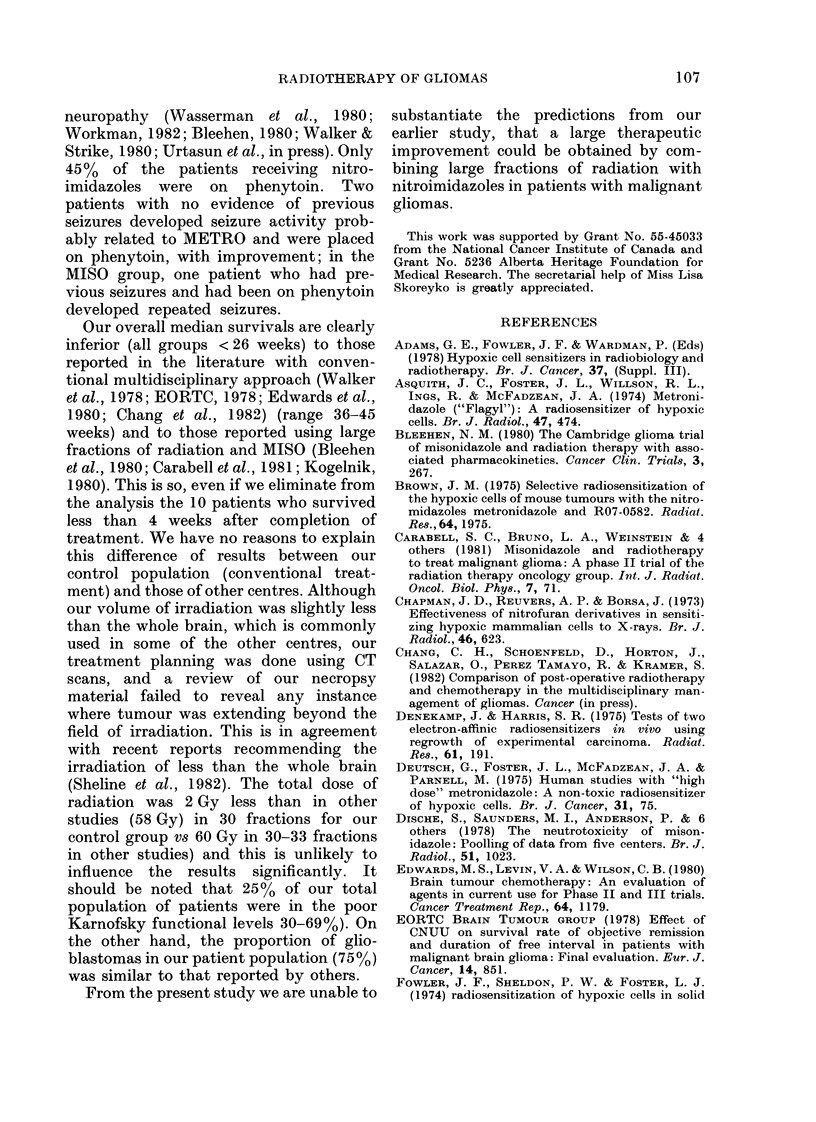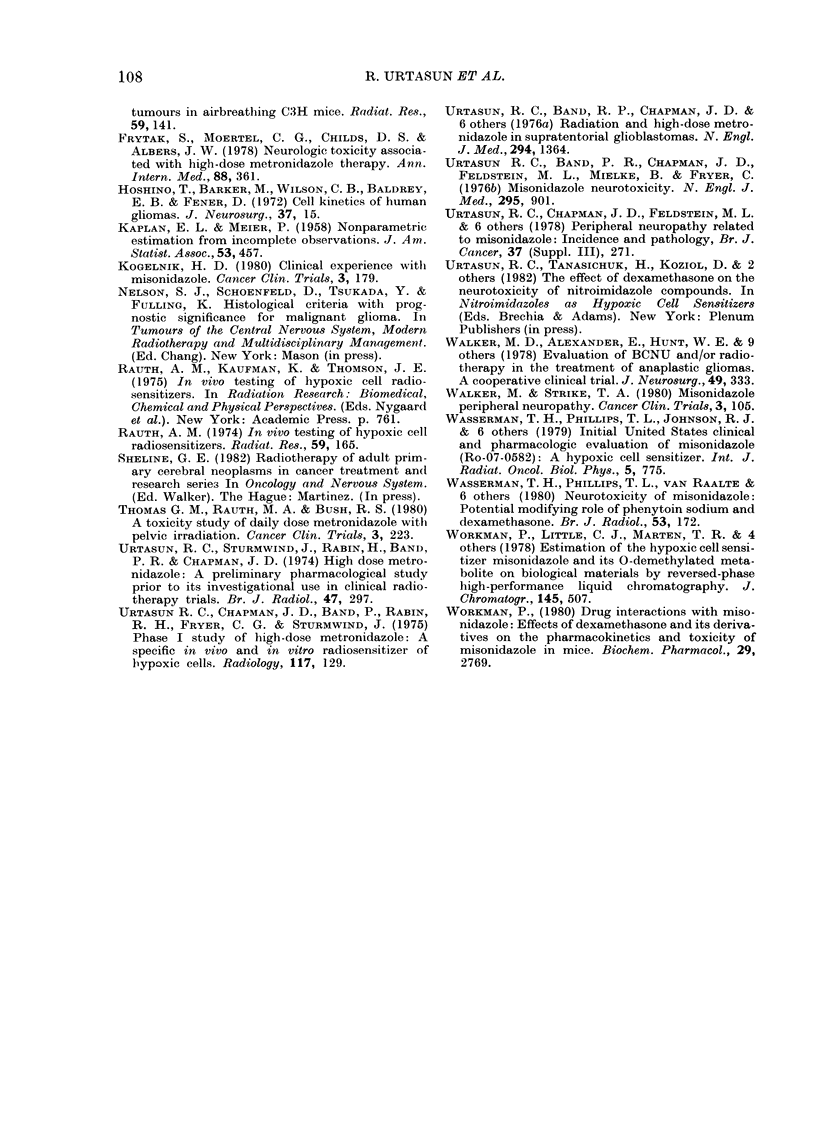# Radiation and nitroimidazoles in supratentorial high grade gliomas: a second clinical trial.

**DOI:** 10.1038/bjc.1982.171

**Published:** 1982-07

**Authors:** R. Urtasun, M. L. Feldstein, J. Partington, H. Tanasichuk, J. D. Miller, D. B. Russell, O. Agboola, B. Mielke

## Abstract

As a continuation of a previous controlled trial using "high-dose" metronidazole as a specific sensitizer of hypoxic cells, we used a more efficient nitroimidazole derivative (misonidazole, MISO) in combination with higher doses of radiation in patients with supratentorial high-grade astrocytomas. Sixty-six patients were stratified according to functional level and histological grading, and randomly allocated within 2 weeks of operation of 1 of 3 therapeutic groups: 1, conventional radiation alone; 2, large fractions of radiation with high-dose metronidazole; and 3, radiation as in Group 2 but with equitoxic doses of MISO. We examined survival as the principal end-point of the study. Neither by increasing the dose of radiation over the previous study, nor by using a more efficient sensitizer, were we able to improve survival over the current conventional daily fractionated radiation.


*Br. J. Cancer* (1982) **46**, 101

### RADIATION AND NITROIMIDAZOLES IN SUPRATENTORIAL HIGH GRADE GLIOMAS: A SECOND CLINICAL TRIAL

R. URTASUN*, M. L. FELDSTEIN†, J. PARTINGTON*, H. TANASICHUK*, J. D. R. MILLER*, D. B. RUSSELL*, O. AGBOOLA* AND B. MIELKE*

*From the *Departments of Radiology and Pathology and Division of Oncology, University of Alberta and Cross Cancer Institute, Edmonton, Alberta, Canada, and †Sydney Farber Cancer Institute, Boston, Massachusetts, U.S.A.*

Received 26 February 1982 Accepted 12 March 1982

**Summary.— As a continuation of a previous controlled trial using "high-dose" metronidazole as a specific sensitizer of hypoxic cells, we used a more efficient nitroimidazole derivative (misonidazole, MISO) in combination with higher doses of radiation in patients with supratentorial high-grade astrocytomas. Sixty-six patients were stratified according to functional level and histological grading, and randomly allocated within 2 weeks of operation to 1 of 3 therapeutic groups: 1, conventional radiation alone; 2, large fractions of radiation with high-dose metronidazole; and 3, radiation as in Group 2 but with equitoxic doses of MISO.**

**We examined survival as the principal end-point of the study.**

**Neither by increasing the dose of radiation over the previous study, nor by using a more efficient sensitizer, were we able to improve survival over the current conventional daily fractionated radiation.**

We HAVE SHOWN in a previous clinical controlled study (Urtasun *et al.*, 1976*a*) that the 5-nitroimidazole compound metronidazole, in combination with external radiation, improved the time to tumour regrowth in patients with supratentorial glioblastomas. This was observed under the strict limitations of the study design, by which both the control and the experimental groups received less than optimal scheduling of radiation and relatively low total tumour dose of radiation. The next logical step was to proceed with another study, in order to compare the best currently available treatment to the unconventional large-fraction radiation (proved to be successful when combined with metronidazole) but combined with a more efficient radiosensitizer.

Nitroimidazole compounds are known to radiosensitize mammalian cells selectively under acutely hypoxic *in vitro* conditions (Chapman *et al.*, 1973; Asquith *et al.*, 1974) and also to radiosensitize hypoxic cells from various animal tumours (Rauth, 1974). Metronidazole was chosen in 1973 for clinical studies as a radiosensitizer, and its toxicity and pharmacological properties at oral high doses has been previously reported (Urtasun *et al.*, 1974, 1975; Deutsch *et al.*, 1975). Misonidazole, a 2-nitroimidazole derivative (1-(2-nitroimidazol-1-yl)-3-methoxypropan-2-ol; Ro-070582; NSC-261037, MISO) has been shown to be a more efficient radiosensitizer of hypoxic mammalian tumour cells, both *in vitro* and *in vivo* (Adams *et al.*, 1978; Brown, 1975; Denekamp *et al.*, 1975; Fowler *et al.*, 1974; Rauth *et al.*, 1975). Both drugs are known to produce CNS and peripheral neurotoxicity as major side effects when used at the high doses necessary to produce radiosensitization, and this has become the dose-limiting

Correspondence to: Dr R. C. Urtasun, 11560 University Avenue, Edmonton, Alberta T6G 1Z2.

factor (Urtasun *et al.*, 1976*b*, 1978; Dishe *et al.*, 1978; Wasserman *et al.*, 1979; Thomas *et al.*, 1980; Frytak *et al.*, 1978).

As in the previous study, we chose patients with glioblastoma multiforme, primarily because of the high rate of local radiation failure, and the small therapeutic gains obtained on combining radiation with currently available cancer chemotherapy agents (Walker *et al.*, 1978; EORTC, 1978; Edwards *et al.*, 1980; Chang *et al.*, 1982) and secondarily because of the characteristic of glioblastoma to form multiple areas of necrosis surrounded by non-proliferating tumour cells, which are assumed to be hypoxic and radioresistant (Hoshino *et al.*, 1972).

In the present study, we have included patients with astrocytoma with anaplastic foci (AAF, Grade III astrocytoma), who comprised 25% of all patients entered in our study. This group of patients has a more favourable prognosis than patients with glioblastomas (Nelson *et al.*, in press) and therefore all patients were stratified according to the histological type. They were also stratified by the functional neurological status.

The therapeutic results with supratentorial high-grade astrocytomas have been poor, though small gains have been made in the past 5 years, by combining chemotherapy and radiation to achieve median survivals of 36–42 weeks when all histological types are considered (glioblastomas as well as AAF; Walker *et al.*, 1978; EORTC, 1978; Edwards *et al.*, 1980; Chang *et al.*, 1982).

### MATERIALS AND METHODS

The centre where the study was conducted serves a population of 1 million. All patients with brain tumours arising from this population are referred to this centre. A neuropathologist assigned to the study reviews and confirms all the pathological slides. Only tumours showing the characteristics of glioblastoma multiforme or astrocytoma with anaplastic foci (AAF) were included. All patients were treated by 1 radiation oncologist. Ten to 14 days after operation, and before randomization, we stratified the patients according to functional Karnofsky status (over 70%, Group A or 30–69%, Group B) and to histological type (glioblastoma *vs* AAF). At the time of entering the study, 77% were considered as Group A and 23% as Group B. Seventy-five per cent were glioblastomas and 25% AAF. Patients were then randomized to receive either conventional radiation alone, metronidazole plus radiation, or MISO plus radiation.

We chose length of remission and patient survival as the end-points, and used CT scans, clinical performance, and dexamethasone requirements as criteria to establish tumour relapse.

*Treatment groups*

*Group* 1.—Radiation alone. We treated the patients with megavoltage $\gamma$-radiation from a cobalt$^{-60}$ source, delivering a total mid-plane tumour dose of 58 Gy in 30 daily fractions, 5 days per weeks in an overall time of 6 weeks, using large parallel opposed fields including 2/3 of the brain volume (tumour plus generous margin of normal brain, in order to cover any possible microscopic extensions).

*Group* 2.—Radiation plus metronidazole (METRO). We used the same source and the same volume and technique of radiation, but the treatments were delivered in 9 large fractions of 4·33 Gy 3 times weekly in an overall time of 3 weeks.

METRO* was given orally at a dose of 6 g/m$^2$ 4 h before each radiaton fraction in the form of 500mg capsules.

*Group* 3.—Radiation plus MISO. The radiation treatment was delivered as described in Group 2. MISO† was given in the form of 500mg capsules, administered by mouth at a dose of 1·25 g/m$^2$ 4 h before each radiation fraction.

All patients receiving the nitroimidazole compounds were restricted to clear fluids by mouth for 7 h before drug administration, to avoid interference with drug absorption. Prochlorperazine (Stemetil), 10 mg by mouth, was administered $\frac{1}{2}$ h before drug ingestion as an antiemetic. All patients were placed on

*Kindly supplied by Poulenc, Montreal, Canada.

†Kindly supplied by Hoffman LaRoche, Montreal, Canada.

TABLE I.—*Patient composition of Group I—conventional radiation (control), Group II—Metronidazole plus radiation and Group III—Misonidazole plus radiation*

| | Group I | Group II | Group III |
|---|---|---|---|
| Age (Mean ± s.d.) | 55·4 ± 10·99 | 56 ± 9·62 | 59·37 ± 8·79 |
| No. | 19 | 17 | 23 |
| Surgery (%) | | | |
| Subtotal | 19·8 | 12·5 | 30·3 |
| Biopsy only | 10·1 | 17·8 | 8·9 |
| Functional status (%) | | | |
| Karnofsky > 70 | 21·4 | 25 | 30·3 |
| Karnofsky 30–69 | 8·9 | 5·3 | 8·9 |
| AAF | 7·1 | 8·9 | 8·9 |
| Glioblastoma | 23·2 | 21·4 | 30·3 |
| Tumour location (%) | | | |
| Parietal | 12·5 | 7·1 | 23·2 |
| Other | 17·8 | 23·2 | 16·0 |

dexamethasone, (Decadron) 9–16 mg daily, at the beginning and for the duration of treatment and then reduced slowly.

CT scans of the brain were performed before and after operation, mid-way through treatments and at monthly intervals until tumour regrowth. Tumour volume, mid-line shift, degree of tumour augmentation and ventricular dilatation were entered in a computer programme for analysis. We considered a patient to have achieved a complete response when there was complete disappearance of direct visual evidence of tumour and absence of mass effect on CT scan, while showing improvement of all clinical neurological signs and symptoms and being off dexamethasone. A partial response was when on CT scan there was over 50% reduction of tumour volume and improvement of mass effect, while showing improvement of neurological signs and symptoms and on decreasing doses of dexamethasone. We considered a patient in relapse when there was CT-scan evidence of increased tumour volume (tumour enhancement peritumoral oedema) while showing a worsening in clinical status (drop of 15% or more in the Karnofsky scale) and requiring doses of dexamethasone.

The study began in August 1976, when 66 patients entered the study and 59 fulfilled the protocol requirements and formed the basis of this report. Five patients were ineligible because of wrong histology, and 2 refused to begin treatment. Table I shows group comparability according to age, histological type, functional status and tumour location.

At the time of documented relapse, we started the patients on CCNU (a nitrosurea compound) at the single dose of 80 mg/m$^2$ orally every 4–5 weeks, provided the WBC was > 4000 and platelet count > $10^5$. The dose was halved if the WBC was 2000–4000 or platelet count 7–10 × $10^4$. Treatments were temporarily discontinued when either the WBC was < 2000 and platelet count < 7 × $10^4$, or there was evidence of pulmonary toxicity.

We measured concentrations of the nitroimidazole compounds in blood at the time of irradiation (4 h after drug ingestion) and whenever possible a 24h drug-kinetic study in blood was performed at the beginning of the 3-week treatment. Measurements used the high-performance liquid chromatography technique of Workman *et al.* (1978).

Before the patients were entered into the study, normal liver-function and renal-function tests and normal peripheral haematological values were required. These tests included blood determinations of alkaline phosphatase, serum glutamic oxalacetic transaminase, bilirubin, blood urea nitrogen, creatinine, uric acid, WBC counts, platelet count, haemoglobin and haematocrit. All tests were done twice weekly during administration of the nitroimidazole compounds, and thereafter at each follow-up.

Whenever possible, necropsies were performed and the brain specimen referred to the neuropathologist assigned to the study.

### RESULTS

Six patients, 2 on each arm, had major protocol violations because of refusal to receive CCNU at the time of tumour relapse. Ten patients required dose modifications of the nitroimidazoles, due mainly

TABLE II.—*Comparative toxicity to treatment in the 3 experimental arms*

| | Group I Control (19) | Group II METRO (17) | Group III MISO (23) |
|---|---|---|---|
| Nausea and vomiting | 1 | 8 | 5 |
| Peripheral neuropathy | 0 | 0 | 0 |
| CNS symptoms* | 8 | 5 | 3 |
| Ototoxicity | 0 | 1 | 2 |
| Hypersensitivity (dermatitis) | 1† | 1 | 3 |
| Dose modifications | 0 | 8 | 2 |

* Include ataxia, shuffling gait, tremors and somnolence.
† Dermatitis related to Dilantin.

TABLE III.—*Tumour response rates* 1 *month after-treatment, using CT scan, clinical evaluation and requirement for Dexamethasone*

| | Group I (19) | | Group II (17) | | Group III (24) | |
|---|---|---|---|---|---|---|
| | No. | % | No. | % | No. | % |
| Complete | 0 | 0 | 0 | 0 | 0 | 0 |
| Partial | 3 | 16 | 2 | 12 | 5 | 21 |
| No change | 9 | 47 | 10 | 58 | 11 | 46 |
| Progression | 7 | 37 | 5 | 29 | 8 | 33 |
| Overall response | 3 | 16 | 2 | 12 | 5 | 21 |

to acute gastrointestinal (GI) toxicity, 8 in the METRO group and 2 in the MISO group. All these 16 patients are included in the final evaluation.

Ten patients died during the first 4 weeks after completion of treatment; 2 in Group 1, and 4 in Groups 2 and 3; 1 in each group, due to causes unrelated to the treatment or to the tumour (myocardial infarction and pulmonary emboli) and the rest due to tumour progression and gross cerebral oedema. One patient in Group 2 was in the favourable histological and functional groups (AFF and Karnofsky over 70%), a second patient was also an AAF but below 60% on the Karnofsky status. A third patient was lost to follow-up at 60 days and therefore considered dead. In Group 3, 3 patients had glioblastomas with 2 of them in the favourable, and 1 in the unfavourable functional group. All 10 patients are included in the study for the final survival analysis.

Of the 59 evaluable patients, 75% were glioblastomas and 25% were considered in the unfavourable functional group. The mean age for the entire population of patients was 58 years. Ten, 5 and 4% of patients in Groups 1, 2 and 3, respectively, were less than 40 years old and 36, 35 and 50% respectively were over 60 years old.

METRO toxicity consisted mainly of nausea and vomiting and CNS symptoms of encephalopathy (ataxia, shuffling gait, somnolence and seizure activity: Table II).

The mean ($\pm$s.d.) plasma concentrations at 4 h after ingestion was $165 \pm 49{\cdot}4$ $\mu$g/ml (range 24–356) for METRO and $38{\cdot}8 \pm 16{\cdot}3$ $\mu$g/ml (range 8–145) for MISO. Plasma half-life was $7{\cdot}8 \pm 3{\cdot}4$ h in 14 patients taking METRO and $10 \pm 3{\cdot}8$ h in 13 patients taking MISO.

The overall response rate at 1 month after treatment was 16% for the control group, 12% for the METRO group and 21% for the MISO group (Table III). There were no complete responders and all 3 groups had similar numbers of partial response.

The observed median length of remission (first day of treatment to relapse) was 175 days for Group 1, 107 days for Group 2 and 157 days for Group 3. No statistical differences among the 3 arms were found (log-rank $P = 0{\cdot}10$). The Kaplan–Meier survival plots (Kaplan, 1958) shown

FIGURE.—Kaplan–Meier survival plots showing the probability of survival with time for patients. There was no difference between the 3 curves.

| Group | Alive | Dead | Total | Median | |
|---|---|---|---|---|---|
| ■ I | 2 | 17 | 19 | 180·7 | 26 weeks |
| ▲ II | 3 | 14 | 17 | 135·7 | 19 weeks |
| ● III | 1 | 22 | 23 | 191·4 | 27 weeks |

Wilcoxon–Gehan, $P = 0\cdot87$.

Log-rank test, $P = 0\cdot99$.

in the Figure, indicate no differences between the 3 survival curves, using the Wilcoxon–Gehan test statistic ($P = 0{\cdot}87$); and the log-rank test ($P = 0{\cdot}99$) confirms the visual impression that there were no differences between the 3 groups. The median survival was 26 weeks for Group 1, 19 weeks for Group 2, and 27 weeks for Group 3. Survival at one year was 16%, 6% and 25% for each group respectively. Analysis of the 1-year survivors on Group 3 reveals 3 patients with glioblastomas and 2 with AAF. All 5 were in the favourable functional group, and the mean age (58) was similar to that of the whole population in the study (15% < 40 years and 65% > 60 years).

Analysis of the whole group of 1-year survival revealed that 50% were glioblastomas (*vs* 75% for all patients) and over 95% were in the favourable functional group.

Our necropsy rate was 30%. Residual tumour was present in all specimens. There were no instances of tumour extending beyond the volume of radiation, either by direct extension or by intraventricular seeding. All had diffuse cerebral oedema plus gross residual mass composed of tumour tissue and necrotic material. We were unable to find any areas of demyelination, suggesting normal-tissue damage by radiation in those patients receiving concomitant nitromidazoles and radiation.

### *Drug toxicity*

Nausea and vomiting, as well as CNS symptoms, were more prevalent in the patients treated with METRO (Table II) and more patients in this group required drug modification (50% *vs* 20% for MISO). There was no evidence of peripheral neuropathy, and there were no short-term changes in the renal- or liver-function test and no changes in the peripheral-blood values, suggesting marrow toxicity.

CCNU toxicity consisted of moderate leucopenia and thrombocytopenia, necessitating occasional drug adjustments. We were unable to detect any instance of pulmonary toxicity. Some of the patients (10%) developed a reversible moderate fall in haemoglobin levels.

### DISCUSSION

On completing our previous study on the use of radiation and METRO in glioblastomas (Urtasun *et al.*, 1976*a*) we concluded that further clinical studies were warranted in view of the observed positive results.

We chose to continue the work with this type of tumour by increasing the dose of radiation per fraction and by using a more effective sensitizer of hypoxic tumour cells. We administered the new radiosensitizer MISO to our patients at a total dose that we considered equitoxic to the previously used METRO ($11{\cdot}25\ g/m^2$ *vs* $54\ g/m^2$ respectively). The maximum total dose of MISO, beyond which there is an unacceptably high incidence of neurotoxicity, is $12 g/m^2$ (Dishe *et al.*, 1978; Wasserman *et al.*, 1979). As in the previous study, we used the same unconventional large fractions of radiation (3 times per week) combined with relatively large doses of the sensitizer, because of inference from *in vitro* studies and animal tumour models then available, that a larger radiosensitization would probably be seen with this approach. We wanted to compare our experimental approach to the currently best accepted method of treating glioblastomas; therefore a control group of patients from the same population was treated at our institution with daily conventional fractionation. All patients received systemic chemotherapy in the form of CCNU at the time of tumour progression. We have found no statistical difference in the median survival time between the 3 groups of patients (Figure) indicating that the combination of radiation at large doses per fraction with either METRO or MISO offers no clear advantage over conventional daily fractionated radiotherapy alone.

This was also seen in the patients in whom we measured the length of remission, though using this end-point the number of patients was considerably reduced because about half of the patients failed to obtain a measurable response.

It should be noted that in our previous publication (Urtasun *et al.*, 1976*a*) the Kaplan–Meier survival curves never crossed one another, whereas in the present study they do, and when this happens with small data sets the statistical tests are bound to be inconclusive. Because of this, we are only able to observe that we are seeing no differences in an improvement at a 60–70% level, but might be unable to detect a 20–30% improvement which clinically could be significant.

In comparing the present results with those of the previously reported study (Urtasun *et al.*, 1976*a*) it is also apparent that neither by increasing the size of the radiation fractions (from 3·33 to 4·33 Gy) nor by using a more efficient radiosensitizer (MISO) were we able to improve the results of the original study. The METRO group in the present study appears to be fairing even worse.

A detailed comparison of therapeutic effectiveness (toxicity and survival) among the group of patients treated with nitroimidazole drugs shows that MISO appears to have an advantage over METRO. Not only did the MISO group of patients have a lower incidence of acute GI and CNS toxicity, but there also appears to be a small difference (though not statistically significant) in the 1-year survival and the median survival times. Of the MISO-treated patients, 25% survived over 1 year, against 6% of the METRO group. The median survival time of 27 weeks for the MISO group contrasts with the 19 weeks for the METRO group. It is true, of course, that these differences are observed because of the unexplained poor survival of patients in the METRO-treated group. This contrasts with the 26-weeks median survival in the previously published METRO study, even though a lower dose of radiation was then used. This difference disappears, however, if we use only patients surviving at least 4 weeks after treatment for analysis. Using this criterion, the median survival for the present METRO group is 28 weeks, for the MISO group, 34 weeks, and for the previous METRO study, 26 weeks. The favourable prognostic factors (AAF, Karnofsky over 70% and age below 40 years were equally distributed among the present MISO and METRO groups.

As previously reported, both the histological type and functional status are important prognostic factors (Nelson *et al.*, in press; Sheline *et al.*, 1982). In our study, this was confirmed when the patients surviving more than a year were analysed. Fifty per cent of these patients were AAF (*vs* 25% incidence of the whole study). Similarly, 95% of these patients had a favourable functional status (compared to 78% in the whole study).

Of interest is the fact that we found no peripheral neuropathies secondary to either drug, though we had expected 25–30% in the patients treated with MISO. We assume that this was probably because all patients were on high-dose dexamethasone while receiving these drugs. Dexamethasone concurrently with MISO has been reported to decrease the incidence of MISO-induced peripheral neuropathy (Wasserman *et al.*, 1980; Workman, 1982; Bleehen, 1980; Walker & Strike, 1980; Urtasun *et al.*, in press). Only 45% of the patients receiving nitroimidazoles were on phenytoin. Two patients with no evidence of previous seizures developed seizure activity probably related to METRO and were placed on phenytoin, with improvement; in the MISO group, one patient who had previous seizures and had been on phenytoin developed repeated seizures.

Our overall median survivals are clearly inferior (all groups < 26 weeks) to those reported in the literature with conventional multidisciplinary approach (Walker *et al.*, 1978; EORTC, 1978; Edwards *et al.*, 1980; Chang *et al.*, 1982) (range 36–45 weeks) and to those reported using large fractions of radiation and MISO (Bleehen *et al.*, 1980; Carabell *et al.*, 1981; Kogelnik, 1980). This is so, even if we eliminate from the analysis the 10 patients who survived less than 4 weeks after completion of treatment. We have no reasons to explain this difference of results between our control population (conventional treatment) and those of other centres. Although our volume of irradiation was slightly less than the whole brain, which is commonly used in some of the other centres, our treatment planning was done using CT scans, and a review of our necropsy material failed to reveal any instance where tumour was extending beyond the field of irradiation. This is in agreement with recent reports recommending the irradiation of less than the whole brain (Sheline *et al.*, 1982). The total dose of radiation was 2 Gy less than in other studies (58 Gy) in 30 fractions for our control group *vs* 60 Gy in 30–33 fractions in other studies) and this is unlikely to influence the results significantly. It should be noted that 25% of our total population of patients were in the poor Karnofsky functional levels 30–69%). On the other hand, the proportion of glioblastomas in our patient population (75%) was similar to that reported by others.

From the present study we are unable to substantiate the predictions from our earlier study, that a large therapeutic improvement could be obtained by combining large fractions of radiation with nitroimidazoles in patients with malignant gliomas.

This work was supported by Grant No. 55-45033 from the National Cancer Institute of Canada and Grant No. 5236 Alberta Heritage Foundation for Medical Research. The secretarial help of Miss Lisa Skoreyko is greatly appreciated.

### REFERENCES

Adams, G. E., Fowler, J. F. & Wardman, P. (Eds) (1978) Hypoxic cell sensitizers in radiobiology and radiotherapy. *Br. J. Cancer*, **37**, (Suppl. III).

Asquith, J. C., Foster, J. L., Willson, R. L., Ings, R. & McFadzean, J. A. (1974) Metronidazole ("Flagyl"): A radiosensitizer of hypoxic cells. *Br. J. Radiol.*, **47**, 474.

Bleehen, N. M. (1980) The Cambridge glioma trial of misonidazole and radiation therapy with associated pharmacokinetics. *Cancer Clin. Trials*, **3**, 267.

Brown, J. M. (1975) Selective radiosensitization of the hypoxic cells of mouse tumours with the nitromidazoles metronidazole and R07-0582. *Radiat. Res.*, **64**, 1975.

Carabell, S. C., Bruno, L. A., Weinstein & 4 others (1981) Misonidazole and radiotherapy to treat malignant glioma: A phase II trial of the radiation therapy oncology group. *Int. J. Radiat. Oncol. Biol. Phys.*, **7**, 71.

Chapman, J. D., Reuvers, A. P. & Borsa, J. (1973) Effectiveness of nitrofuran derivatives in sensitizing hypoxic mammalian cells to X-rays. *Br. J. Radiol.*, **46**, 623.

Chang, C. H., Schoenfeld, D., Horton, J., Salazar, O., Perez Tamayo, R. & Kramer, S. (1982) Comparison of post-operative radiotherapy and chemotherapy in the multidisciplinary management of gliomas. *Cancer* (in press).

Denekamp, J. & Harris, S. R. (1975) Tests of two electron-affinic radiosensitizers *in vivo* using regrowth of experimental carcinoma. *Radiat. Res.*, **61**, 191.

Deutsch, G., Foster, J. L., McFadzean, J. A. & Parnell, M. (1975) Human studies with "high dose" metronidazole: A non-toxic radiosensitizer of hypoxic cells. *Br. J. Cancer*, **31**, 75.

Dische, S., Saunders, M. I., Anderson, P. & 6 others (1978) The neutrotoxicity of misonidazole: Poolling of data from five centers. *Br. J. Radiol.*, **51**, 1023.

Edwards, M. S., Levin, V. A. & Wilson, C. B. (1980) Brain tumour chemotherapy: An evaluation of agents in current use for Phase II and III trials. *Cancer Treatment Rep.*, **64**, 1179.

EORTC Brain Tumour group (1978) Effect of CNUU on survival rate of objective remission and duration of free interval in patients with malignant brain glioma: Final evaluation. *Eur. J. Cancer*, **14**, 851.

Fowler, J. F., Sheldon, P. W. & Foster, L. J. (1974) radiosensitization of hypoxic cells in solid tumours in airbreathing C3H mice. *Radiat. Res.*, **59,** 141.

FRYTAK, S., MOERTEL, C. G., CHILDS, D. S. & ALBERS, J. W. (1978) Neurologic toxicity associated with high-dose metronidazole therapy. *Ann. Intern. Med.*, **88,** 361.

HOSHINO, T., BARKER, M., WILSON, C. B., BALDREY, E. B. & FENER, D. (1972) Cell kinetics of human gliomas. *J. Neurosurg.*, **37,** 15.

KAPLAN, E. L. & MEIER, P. (1958) Nonparametric estimation from incomplete observations. *J. Am. Statist. Assoc.*, **53,** 457.

KOGELNIK, H. D. (1980) Clinical experience with misonidazole. *Cancer Clin. Trials*, **3,** 179.

NELSON, S. J., SCHOENFELD, D., TSUKADA, Y. & FULLING, K. Histological criteria with prognostic significance for malignant glioma. In *Tumours of the Central Nervous System, Modern Radiotherapy and Multidisciplinary Management.* (Ed. Chang). New York: Mason (in press).

RAUTH, A. M., KAUFMAN, K. & THOMSON, J. E. (1975) *In vivo* testing of hypoxic cell radiosensitizers. In *Radiation Research: Biomedical, Chemical and Physical Perspectives.* (Eds. Nygaard *et al.*). New York: Academic Press. p. 761.

RAUTH, A. M. (1974) *In vivo* testing of hypoxic cell radiosensitizers. *Radiat. Res.*, **59,** 165.

SHELINE, G. E. (1982) Radiotherapy of adult primary cerebral neoplasms in cancer treatment and research series In *Oncology and Nervous System.* (Ed. Walker). The Hague: Martinez. (In press).

THOMAS G. M., RAUTH, M. A. & BUSH, R. S. (1980) A toxicity study of daily dose metronidazole with pelvic irradiation. *Cancer Clin. Trials*, **3,** 223.

URTASUN, R. C., STURMWIND, J., RABIN, H., BAND, P. R. & CHAPMAN, J. D. (1974) High dose metronidazole: A preliminary pharmacological study prior to its investigational use in clinical radiotherapy trials. *Br. J. Radiol.*, **47,** 297.

URTASUN R. C., CHAPMAN, J. D., BAND, P., RABIN, R. H., FRYER, C. G. & STURMWIND, J. (1975) Phase I study of high-dose metronidazole: A specific *in vivo* and *in vitro* radiosensitizer of hypoxic cells. *Radiology*, **117,** 129.

URTASUN, R. C., BAND, R. P., CHAPMAN, J. D. & 6 others (1976*a*) Radiation and high-dose metronidazole in supratentorial glioblastomas. *N. Engl. J. Med.*, **294,** 1364.

URTASUN R. C., BAND, P. R., CHAPMAN, J. D., FELDSTEIN, M. L., MIELKE, B. & FRYER, C. (1976*b*) Misonidazole neurotoxicity. *N. Engl. J. Med.*, **295,** 901.

URTASUN, R. C., CHAPMAN, J. D., FELDSTEIN, M. L. & 6 others (1978) Peripheral neuropathy related to misonidazole: Incidence and pathology, *Br. J. Cancer*, **37** (Suppl. III), 271.

URTASUN, R. C., TANASICHUK, H., KOZIOL, D. & 2 others (1982) The effect of dexamethasone on the neurotoxicity of nitroimidazole compounds. In *Nitroimidazoles as Hypoxic Cell Sensitizers* (Eds. Brechia & Adams). New York: Plenum Publishers (in press).

WALKER, M. D., ALEXANDER, E., HUNT, W. E. & 9 others (1978) Evaluation of BCNU and/or radiotherapy in the treatment of anaplastic gliomas. A cooperative clinical trial. *J. Neurosurg.*, **49,** 333.

WALKER, M. & STRIKE, T. A. (1980) Misonidazole peripheral neuropathy. *Cancer Clin. Trials*, **3,** 105.

WASSERMAN, T. H., PHILLIPS, T. L., JOHNSON, R. J. & 6 others (1979) Initial United States clinical and pharmacologic evaluation of misonidazole (Ro-07-0582): A hypoxic cell sensitizer. *Int. J. Radiat. Oncol. Biol. Phys.*, **5,** 775.

WASSERMAN, T. H., PHILLIPS, T. L., VAN RAALTE & 6 others (1980) Neurotoxicity of misonidazole: Potential modifying role of phenytoin sodium and dexamethasone. *Br. J. Radiol.*, **53,** 172.

WORKMAN, P., LITTLE, C. J., MARTEN, T. R. & 4 others (1978) Estimation of the hypoxic cell sensitizer misonidazole and its O-demethylated metabolite on biological materials by reversed-phase high-performance liquid chromatography. *J. Chromatogr.*, **145,** 507.

WORKMAN, P., (1980) Drug interactions with misonidazole: Effects of dexamethasone and its derivatives on the pharmacokinetics and toxicity of misonidazole in mice. *Biochem. Pharmacol.*, **29,** 2769.